# Characterization and gene expression patterns analysis implies BSK family genes respond to salinity stress in cotton

**DOI:** 10.3389/fgene.2023.1169104

**Published:** 2023-06-07

**Authors:** Yuqian Lei, Yupeng Cui, Ruifeng Cui, Xiugui Chen, Junjuan Wang, Xuke Lu, Delong Wang, Shuai Wang, Lixue Guo, Yuexin Zhang, Cun Rui, Yapeng Fan, Mingge Han, Lanjie Zhao, Hong Zhang, Xiaoyu Liu, Nan Xu, Jing Wang, Hui Huang, Xixian Feng, Yanlong Xi, Kesong Ni, Menghao Zhang, Tiantian Jiang, Wuwei Ye

**Affiliations:** ^1^ Zhengzhou Research Base, State Key Laboratory of Cotton Biology, School of Agricultural Sciences, Zhengzhou University, Zhengzhou, Henan, China; ^2^ State Key Laboratory of Cotton Biology, Institute of Cotton Research of Chinese Academy of Agricultural Sciences, Anyang, Henan, China; ^3^ Anyang Institute of Technology, Anyang, Henan, China

**Keywords:** BR, BSK, brassinosteroid-signaling kinase, abiotic stress, salt stress

## Abstract

Identification, evolution, and expression patterns of BSK (BR signaling kinase) family genes revealed that BSKs participated in the response of cotton to abiotic stress and maintained the growth of cotton in extreme environment. The steroidal hormone brassinosteroids (BR) play important roles in different plant biological processes. This study focused on BSK which were downstream regulatory element of BR, in order to help to decipher the functions of BSKs genes from cotton on growth development and responses to abiotic stresses and lean the evolutionary relationship of cotton BSKs. BSKs are a class of plant-specific receptor-like cytoplasmic kinases involved in BR signal transduction. In this study, bioinformatics methods were used to identify the cotton BSKs gene family at the cotton genome level, and the gene structure, promoter elements, protein structure and properties, gene expression patterns and candidate interacting proteins were analyzed. In the present study, a total of 152 BSKs were identified by a genome-wide search in four cotton species and other 11 plant species, and phylogenetic analysis revealed three evolutionary clades. It was identified that BSKs contain typical PKc and TPR domains, the N-terminus is composed of extended chains and helical structures. Cotton BSKs genes show different expression patterns in different tissues and organs. The gene promoter contains numerous *cis*-acting elements induced by hormones and abiotic stress, the hormone ABA and Cold-inducing related elements have the highest count, indicating that cotton BSK genes may be regulated by various hormones at different growth stages and involved in the response regulation of cotton to various stresses. The expression analysis of BSKs in cotton showed that the expression levels of *GhBSK06*, *GhBSK10*, *GhBSK21* and *GhBSK24* were significantly increased with salt-inducing. This study is helpful to analyze the function of cotton BSKs genes in growth and development and in response to stress.

## 1 Introduction

With a habit of sessile growth, plants need to evolve a variety of regulatory methods to adapt to changes in the environment and deal with stress. Brassinosteroids (BR) has been reported as an important plant hormone involved in plant cell proliferation, senescence, male sterility, inducing flowering and fruit ripening and resisting abiotic stress (Clouse and Sasse, 1998; Nakaya et al., 2002; Symons et al., 2006; Bajguz and Hayat, 2009; Li et al., 2010; Ye et al., 2010; Fàbregas and Caño-Delgado, 2014). In the BR signaling pathway model that has been explored and established, many regulatory elements play an important role. The upstream elements include BRI (BR insensitive), BKI (BRI kinase inhibitor 1), BAK (BRI associated receptor kinase) and BSK (BR signaling kinase), and the downstream elements are jointly regulated by BIN2 (BR insensitive 2), BSU (BRI1 suppressor), BZR (BR resistant transcription factor) and BES (BRI-EMS suppressor) (Choudhary et al., 2012).

BSK is a class of receptor-like cytoplasmic kinases involved in BR signaling transduction. BSK is a phosphorylated substrate of BRI1, and after activation, it transmits signals downstream and is involved in regulating the expression of BR responsive genes (He et al., 2000). BSKs belong to the RLCK-XII superfamily, and their encoded proteins contain two typical kinase domains, PKc (Putative kinase catalytic) and TPR (Tetra-tri-co peptide repeat domains) domains. PKc can play a role in cell division, proliferation, apoptosis and differentiation, and TPR can mediate the interaction between proteins.

As an important cash crop, cotton inevitably suffers from adversity during the growth process, which affects its yield and quality. Therefore, revealing the components of cotton BR signal and its transduction pathway is of great significance for the response to stress and the regulation of growth and development. There are 12 BSKs in the model plant *Arabidopsis thaliala* (Li et al., 2019). Except for the bsk3-1 mutant which significantly reduced sensitivity to BR, the other single mutants showed no obvious phenotypic changes (Tang et al., 2008). However, quadruple mutants bsk3, 4, 7, 8 and quintuple mutants bsk3, 4, 6, 7, 8 significantly reduced the sensitivity and growth phenotype of BR, indicating that plant BSKs have functional redundancy (Kim et al., 2009; Kim et al., 2011; Sreeramulu et al., 2013). BSKs regulate BR signaling *via* an allosteric mechanism constitutively inactive protein kinases (Grütter et al., 2013).

Recent studies have pointed out that *Arabidopsis BSK1* is physiologically related to pathogen-related molecular patterns PAMP (Pathogen-associated molecular patterns) and FLS2 (Flagellin-sensitive 2), and participates in the positive regulation of PAMP-triggered immunity (PAMP-triggered immunity), preventing the spread of pathogen infection and improving plant disease resistance (Stout et al., 2006; Bent and Mackey, 2007; Schwessinger and Zipfel, 2008; Boller and Felix, 2009; Shi et al., 2013). So far, there have been no reports on the functional studies of cotton BSKs genes in hormone signal transduction, growth and development, and response to stress. The expression level of two *BSKs* in transgenic *ScALDH21* cotton was higher than that of non-transgenic cotton under salt stress (Yang et al., 2023). Overexpression of *BSKs* in crops such as rice and maize has been found to enhance salt tolerance (Li et al., 2022; Liu et al., 2022; Zhang et al., 2022). However, the detailed characterization of BSK family proteins and their important function in plants remains unclear.

In this study, bioinformatics methods were used to analyze the gene structure, evolution, and expression patterns of cotton BSKs gene family. It lays the foundation for the transduction pathway, thereby providing new genetic resources for cotton breeding.

## 2 Materials and methods

### 2.1 Data download

The genome annotation files, protein sequence data, the genome sequence information (fasta format file) and genome gene structure annotation information (gff3 format file) of the four cotton species *Gossypium arboreum* (CRI), *Gossypium raimondii* (JGI), *Gossypium hirsutum* (ZJU) and *Gossypium barbadense* (ZJU) were obtained from Cotton Functional Genomics Database (CottonFGD) (Zhu et al., 2017) (http://www.cottonfgd.org/). The latest Genome annotation files and protein sequence data of other seven species *Arabidopsis thaliana. TAIR10*, *Glycine max_v2.1*, *Oryza sativa. IRGSP-1.0*, *Populus trichocarpa. Pop_tri_v3*, *Theobroma cacao_20110822*, *Vitis vinifera.12X* and *Zea mays. B73_RefGen_v4* were provided by JGI (https://gold.jgi.doe.gov/).

These softwares were used: TBtools (https://github.com/CJ-Chen/TBtools/releases), BLAST+ (https://ftp.ncbi.nlm.nih.gov/blast/executables/blast+/LATEST/), HMMER3.0 (http://www.hmmer.org/) and MEGA7 (https://www.megasoftware.net/). The Hidden Markov Models (HMMs) profile of PF07714 (PKc) and PF07719 (TPR) were downloaded from Pfam (https://pfam.xfam.org/).

### 2.2 Identification of BSK family genes

The BSK family members of *Arabidopsis* have been identified, with a total of 12 members (Li et al., 2019). The protein sequences of the 12 AtBSK family members were downloaded from the Tair website (https://www.arabidopsis.org/) as seed sequences to perform multiple sequence alignments in the plant protein database with BLAST + to identify the BSK family members of *G. hirsutum*, *G. barbadense*, *G. arboreum*, *G. raimondii*, *G. max*, *O. sativa*, *P. trichocarpa*, *T. cacao*, *V. vinifera* and *Z. mays*. The e value was less than 1e-5. The protein sequences of the members were queried with HMMER3.0 to obtain the correct family members (Zhang et al., 2021). TBtools helped to rename all the identified genes according to the gene ID to facilitate subsequent analysis.

### 2.3 Phylogenetic analysis and sequences alignments

In order to explore the evolutionary relationship between BSK family members, it is necessary to submit the protein sequences to MEGA7 (Kumar et al., 2016) to calculate the optimal evolutionary model and to construct a phylogenetic tree using the maximum likelihood method based on this model. The detailed parameters were as follows: maximum likelihood, Bootstrap replications 500, Jones-taylor-Thornton (JTT) model, Gamma distributed with Invariant sites (G + I), No of Discrete Gamma Categories 5, Partial deletion 80 (Huang et al., 2022). The Evolview website (https://www.evolgenius.info/evolview/) was used for visual analysis of the evolutionary tree (Wang et al., 2021).

### 2.4 Conserved protein motifs, gene structure analysis and prediction of subcellular localization

The identified BSK protein sequences of four cotton species were submitted to the Motif Elicitation (MEME) (https://meme-suite.org/) online website to obtain the conserved protein motifs and save the MAST xml file. The nwk file of cotton phylogenetic tree which was construct by MEGA7 was also needed. The MAST xml file, nwk file and gff3 genome file of cotton were placed to TBtools to gain a phylogenetic tree with conserved motifs and gene structures. *BSK* family protein sequences were submitted to the website WoLF PSORT: Protein Subcellular Localization Prediction (https://wolfpsort.hgc.jp/) for subcellular localization prediction.

### 2.5 Collinearity analysis of *BSK* genes in four cotton species

The collinearity between the *BSK* duplicate gene pairs of the four cotton species was analyzed by MCScanX software on the four cotton protein sequence data files, and the graphical results were visualized with TBtools software.

### 2.6 Calculation of selection pressure

The CDS sequences of the *BSK* genes in the four cotton species was downloaded from CottonFGD (http://www.cottonfgd.org/). The homologous gene pairs in the four cotton species from duplicated gene pairs were obtained by TBtools. In order to investigate the selection pressure, we calculated the ratio of the number of non-synonymous substitutions per non-synonymous site to the number of synonymous substitutions per synonymous site (*Ka/Ks*) for duplicated genes.

### 2.7 *Cis*-acting elements and expression pattern analysis

For the analysis of the promoter region of *BSK*, the upstream 2000 bp DNA sequence of *BSK* was downloaded from CottonFGD and uploaded to PlantCare website (http://bioinformatics.psb.ugent.be/webtools/plantcare/html/) to predict the *cis*-acting elements (Wang et al., 2021). The nwk file of BSK phylogenetic tree, *cis*-acting element prediction results and CDS were submitted to TBtools for visual analysis.

For the analysis of the expression pattern (fragments per kilo base of exon per million mapped, FPKM) of BSK family genes, we downloaded RNA-Seq data from GRAND (Hu et al., 2019) (*Gossypium* Resource And Network Database) (http://grand.cricaas.com.cn/home) and draw heatmap based on the expression levels of BSK family genes *via* TBtools. The expression level change of *GhBSKs* under salt stress was shown in bar graph.

## 3 Results

### 3.1 Identification of *BSK* family members

After BLAST + sequence alignment, there are 28, 27, 13 and 15 members of *BSK* gene family in *G. hirsutum*, *G. barbadense*, *G. arboretum* and *G. raimondii*, a total of 83 were identified. The number of BSK members of tetraploid *G. hirsutum* and *G. barbadense* is twice that of diploid *G. arboretum* and *G. raimondii*. This became strong evidence that tetraploid cotton was an allotetraploid formed by hybridizing A-genome diploid and D-genome diploid. As for the other species, *G. max*, *O. sativa*, *P. trichocarpa*, *T. cacao*, *V. vinifera* and *Z. mays* were identified 13, 5, 14, 8, 7 and 10 BSK members. A total of 140 members were identified from the 10 species, and only one complete splice mutant was retained for each genomic locus. The total 140 BSK genes identified in these 10 species were sorted by gene ID and renamed with TBtools as *GhBSK01* - *GhBSK28*, *GbBSK01* - *GbBSK27*, *GaBSK01* - *GaBSK13*, *GrBSK01* - *GrBSK15*, *GmBSK01* - *GmBSK13*, *OsBSK01* - *OsBSK05*, *PtBSK01* - *PtBSK14, TcBSK01* - *TcBSK08*, *VvBSK01* - *VvBSK07* and *ZmBSK01* - *ZmBSK10* (Supplementary Table S1). The names of *AtBSK* family members were consistent with Tair website. The *BSKs* of tetraploid cotton has more *BSK* members than diploid plants such as rice and grapes, indicating that *BSK* has gene duplication in evolution.

### 3.2 Phylogenetic analysis and evolution analysis

**FIGURE 1:**
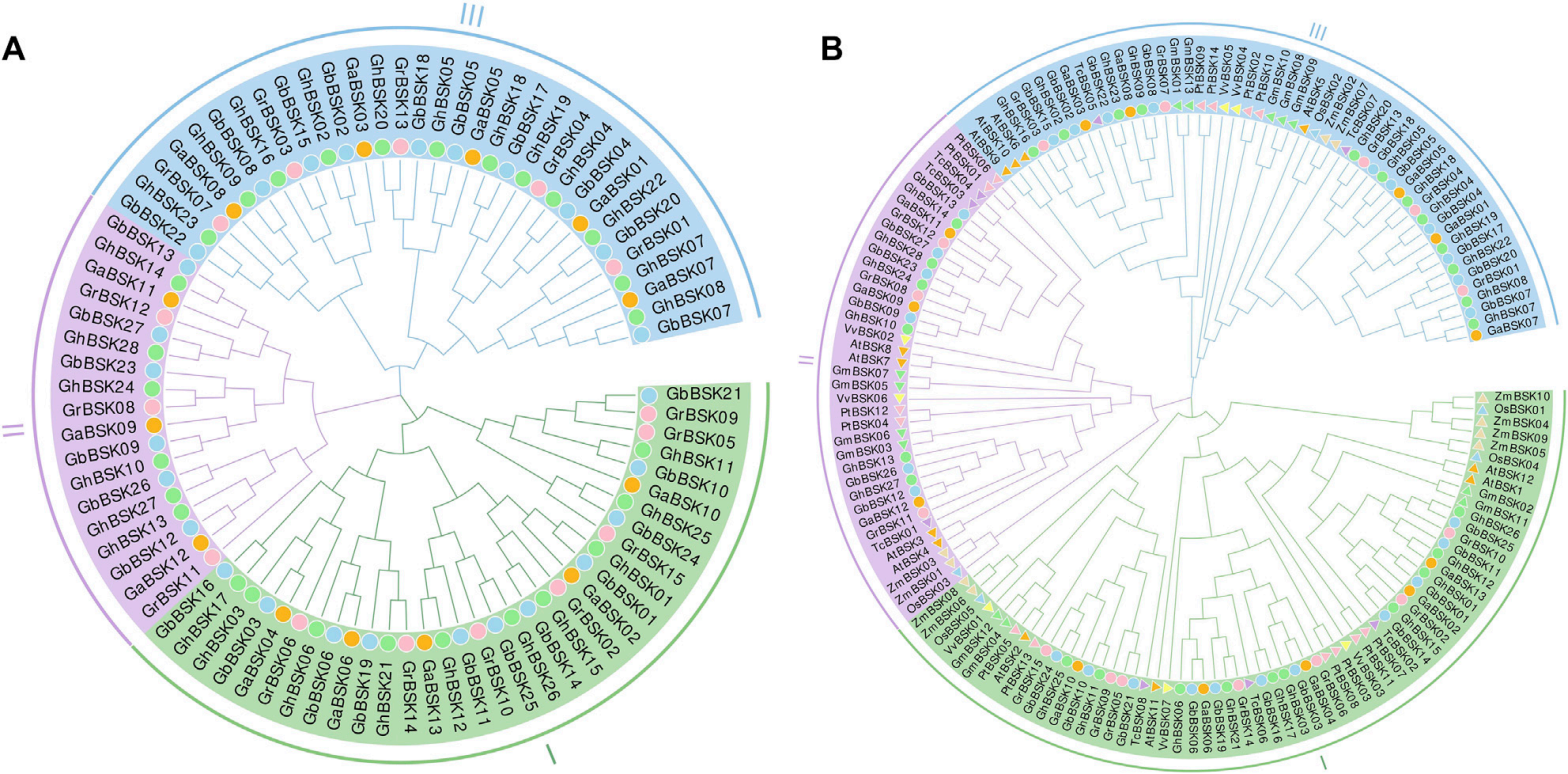
Phylogenetic analysis of plant BSKs. **(A)** The ML tree of identified BSKs from four cotton species. **(B)** The ML tree of BSKs from four cotton species, *Arabidopsis thaliana* and other six plant species.

The phylogenetic relationship between BSK proteins is shown in the phylogenetic tree shown in Figure 1. The phylogenetic tree shows that BSKs have three distinct evolutionary branches, classified as BSK I - BSK III. ZmBSKs and OsBSKs proteins were more closely related. The number of different classes of BSKs from different species were shown in Table 1. There were more BSK I and BSK III present in cotton, twice as much as BSK II. *Arabidopsis thaliana*, *G. max*, *V. vinifera* and *T. cacao* have an even distribution. *O. sativa*, *P. trichocarpa* and *Z. mays* have more BSK I, but less BSK II and BSK III.

**TABLE 1:** Distribution of BSKs in different species.

Species	BSK I	BSK II	BSK III	Total
*G. hirsutum*	10	6	12	28
*G. barbadens*	11	6	10	27
*G. arboreum*	5	3	5	13
*G. raimondii*	7	3	5	15
*A. thaliana*	4	4	4	12
*G. max*	4	4	5	13
*O. sativa*	3	1	1	5
*P. trichocarpc*	6	4	4	14
*T. cacao*	3	3	2	8
*V. vinifera*	3	2	2	7
*Z. mays*	6	2	2	10

### 3.3 Chromosomal localization of four cotton species

**FIGURE 2:**
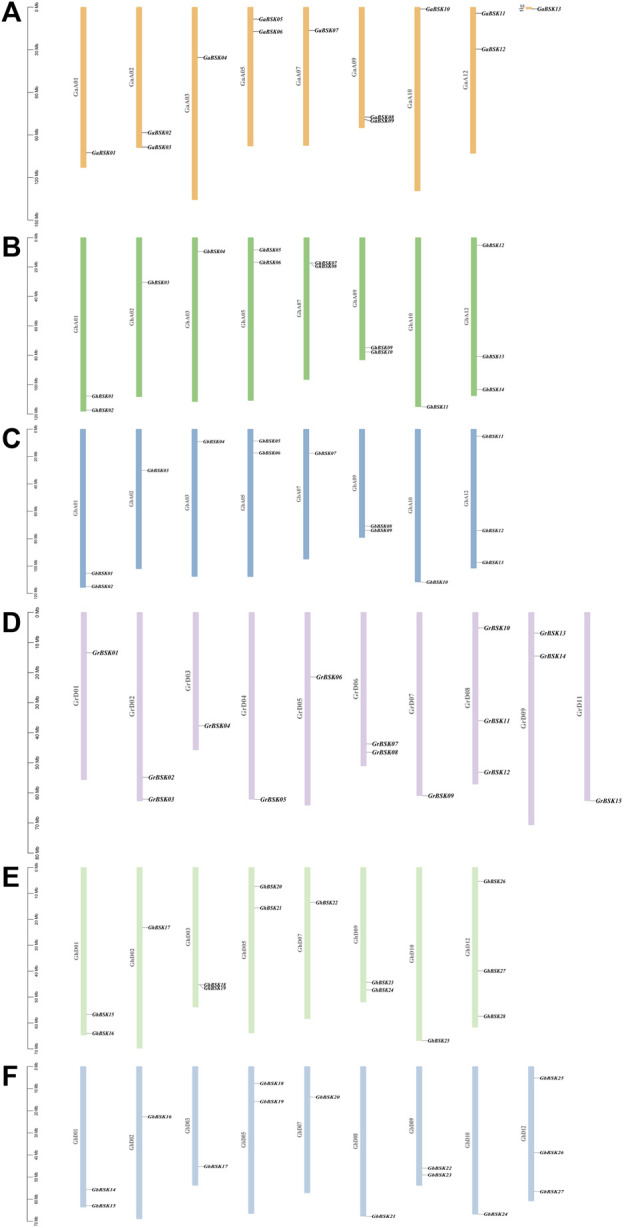
Chromosomal locations of the four cotton species *BSK* gene family members. The scale on the left is in mega-bases. The gene ID on the right side of each chromosome corresponds to the location of each BSK gene. **(A)** *G. arboreum* A-genome “Ga”, **(B)** *G. hirsutum* At-sub genome “GhA”, **(C)** *G. barbadense* At-sub genome “GbA”, **(D)** *G. raimondii* D-genome “Gr”, **(E)** *G. hirsutum* Dt-sub genome “GhD”, **(F)** *G. barbadense* Dt-sub genome “GbD”.

Chromosome distribution maps show the distribution of cotton BSK members on chromosomes in detail, and counted the number of genes on each chromosome (Table 2). The distribution in *G. hirsutum*, *G. barbadense*, and *G. arboreum* is highly conserved, and the fourth, sixth, eighth (excepted GbD), 11th and 13th chromosomes in each group of chromosomes have no BSK distribution (Figure 2). While *G. raimondii* is different from the other three species. There is no gene distribution on the 10th, 12th, and 13th chromosomes. Only one BSK gene of *G. arboretum* is located on *Tig*, which means the evolution of BSK gene family is mature.

**TABLE 2:** Number distribution of cotton BSKs in different chromosome.

Chromsomes	GhA	GhD	GbA	GbD	GaA	GrD
Chir 1	2	2	2	2	1	1
Chir 2	1	1	1	1	2	2
Chir 3	1	2	1	1	1	1
Chir 4						1
Chir 5	2	2	2	2	2	1
Chir 6						2
Chir 7	2	1	1	2	1	1
Chir 8						3
Chir 9	2	2	2	2	2	2
Chir 10	1	1	1	1	1	
Chir 11						1
Chir12	3	3	3	3	2	
Chir13						
Tig					1	
Total	14	14	13	14	13	15

### 3.4 Conserved protein motifs, gene structure analysis and prediction of subcellular localization

**FIGURE 3:**
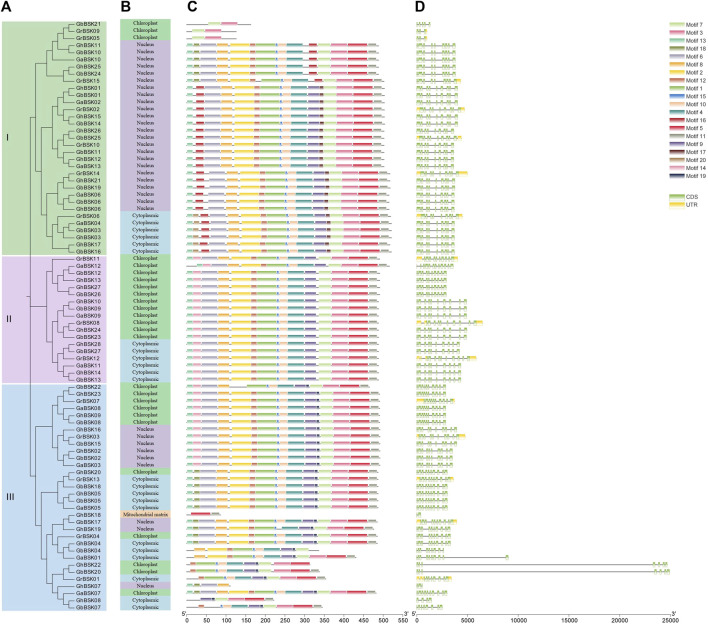
Conserved domain recognition and gene structure analysis of *GhBSKs* and *GbBSKs*. **(A)** Phylogenetic tree of *GhBSKs* and *GbBSKs*, **(B)** Prediction of subcellular localization of *GhBSKs* and *GbBSKs*, **(C)** Conserved motifs of GhBSKs and GbBSKs proteins, **(D)** Exon-intron structures of *GhBSKs* and *GbBSKs*.

Motif architecture, exon-intron structure analysis was performed on 83 BSKs of cotton, and their evolutionary relationships were visualized and drawn into a map (Figure 3). *GhBSK08*, *GhBSK18* and *GbBSK07* only lack PKc, and *GhBSK19* and *GhBSK22* only lack TPR. Both domains are missing from *GhBSK07*, *GbBSK21*, *GrBSK05*, and *GrBSK09*. The remaining 74 BSKs contain two domains. *GbBSK22* has two PKcs. The remaining 74 BSKs contain two domains, and their motifs are well conserved. Some BSK IIIs only retain a small number of motifs, which may be very critical to the function of BSK. Further, BSKs in the same clade were always predicted to have the same subcellular localization.

### 3.5 Gene duplication and collinearity analysis

**FIGURE 4:**
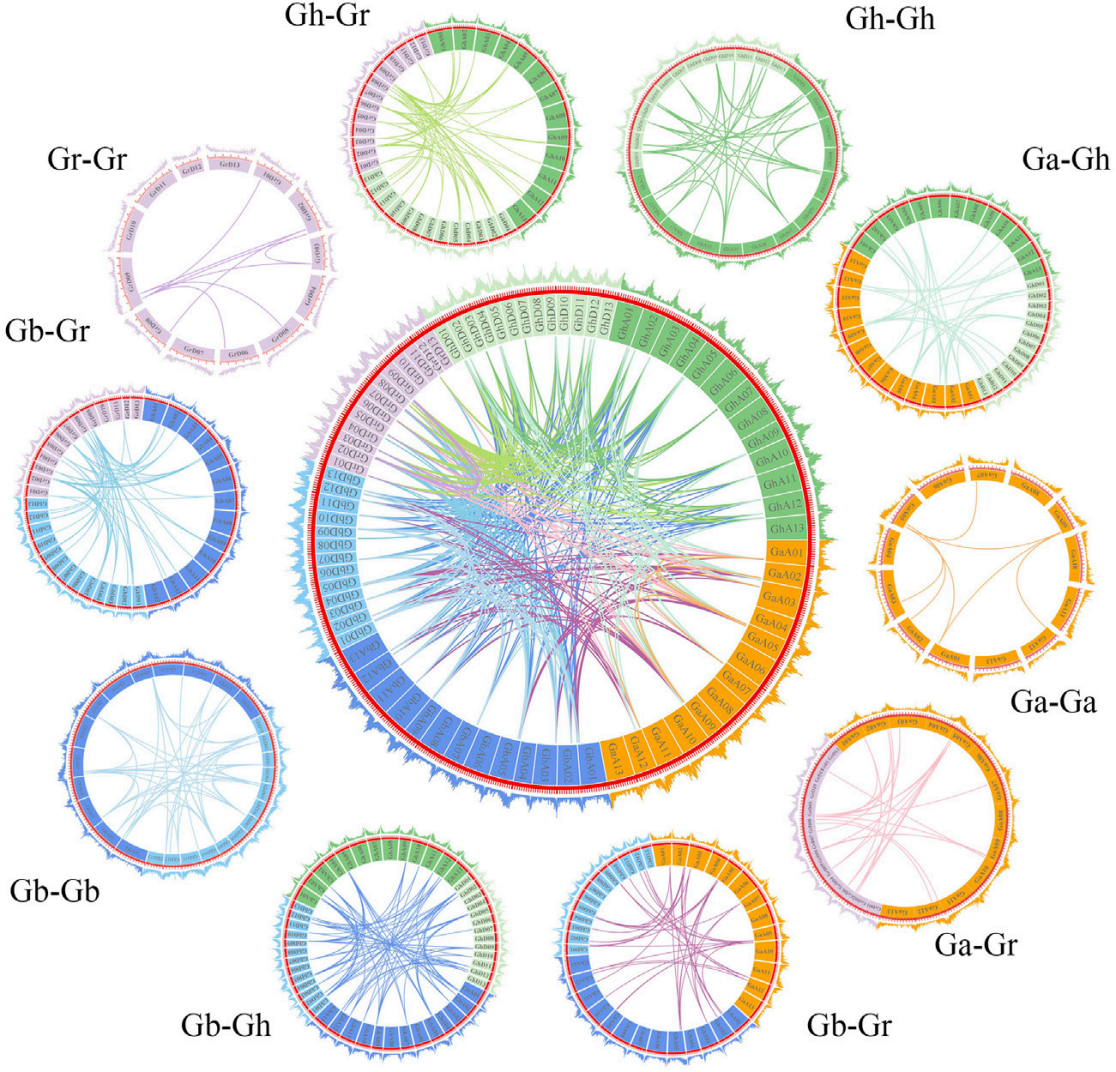
The collinearity relationship of repeated BSK gene pairs in the four cotton species of *Gossypium hirsutum*, *Gossypium barbadense*, *Gossypium arboreum* and *Gossypium raimondii*. The inner ring uses different colors to represent the chromosomes of different cotton species, the inner lines represent the collinearity of BSK genes, the outer ring represents the density of genes on the chromosome.

To explore the evolutionary process of BSK genes, MCscanX was used to analyze the gene duplication patterns of four cotton species and conduct genetic correlation analysis (Figure 4). A total of 385 gene pairs of genome-wide duplication were identified from 4 cotton species, of which 94 were segmental duplication and no tandem duplication was found. Based on this result, it is speculated that parallel homologous genes are generated through whole-genome duplication and segmental duplication and whole-genome duplication is an important driving force for species differentiation. Gh and Gb collinearity gene pairs are the most among the 10 groups, with 81 pairs, while Ga and Ga have the least, with only 7 pairs, which is in line with the comparison of the number of diploid and tetraploid genes. The other collinear pairs of Ga-Gb, Ga-Gh, Ga-Gr, Gb-Gb, Gb-Gr, Gh-Gh, Gh-Gr and Gr-Gr were 39, 35, 22, 41, 81, 59, 38, 55 and 8.

### 3.6 Selection pressure Ka/Ks is analyzed

**FIGURE 5:**
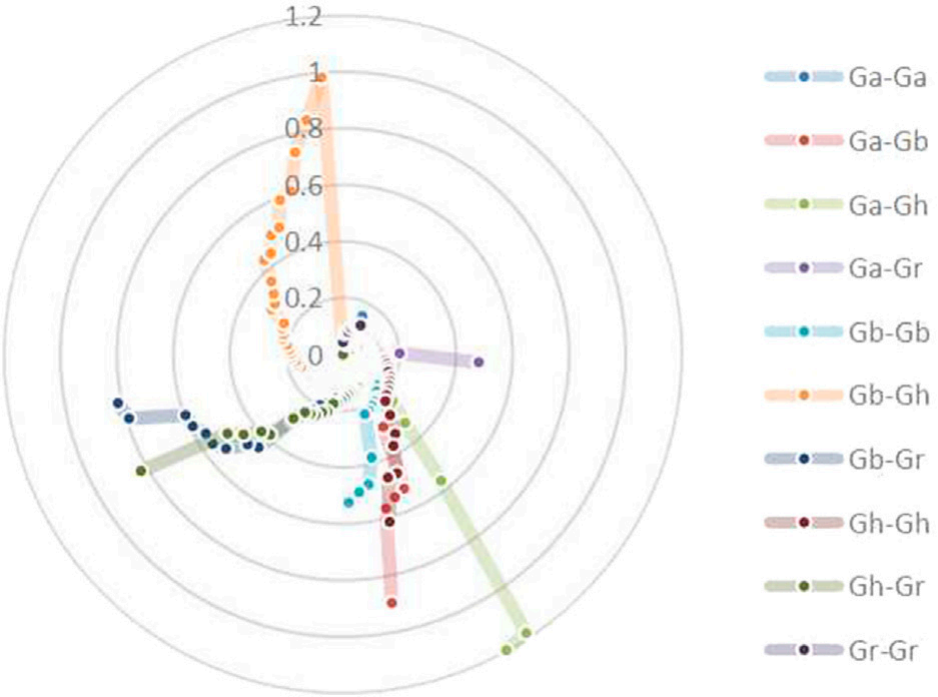
Analysis of non-synonymous (*Ka*) to synonymous (*Ks*) ratio.

To investigate the relationship between Darwinian positive selection and BSK gene duplication, the non-synonymous levels (Ka) and synonymous levels (Ks) were calculated for 385 duplicated gene pairs in 10 combinations from four cotton species. These combinations include Ga-Ga, Ga-Gb, Ga-Gh, Ga-Gr, Gb-Gb, Gb-Gh, Gb-Gr, Gh-Gh, Gh-Gr, Gr-Gr. Inference of selection pressure for duplicate gene pairs was based on Ka/Ks values. It is generally acknowledged that Ka/Ks = 1 indicates neutral selection (pseudogenes), Ka/Ks < 1 indicates purification or negative selection, which means that the gene evolution has a trend towards purification, and Ka/Ks > 1 indicates positive selection. Gene pairs with Ka/Ks > 1 appeared in all groups except Ga-Ga and Gr-Gr, indicating that these genes evolved faster. Most gene pairs have Ka/Ks values within 1, centered between 0 and 0.5 (Figure 5; Supplementary Table S2).

### 3.7 *Cis*-acting elements and expression pattern analysis

**FIGURE 6:**
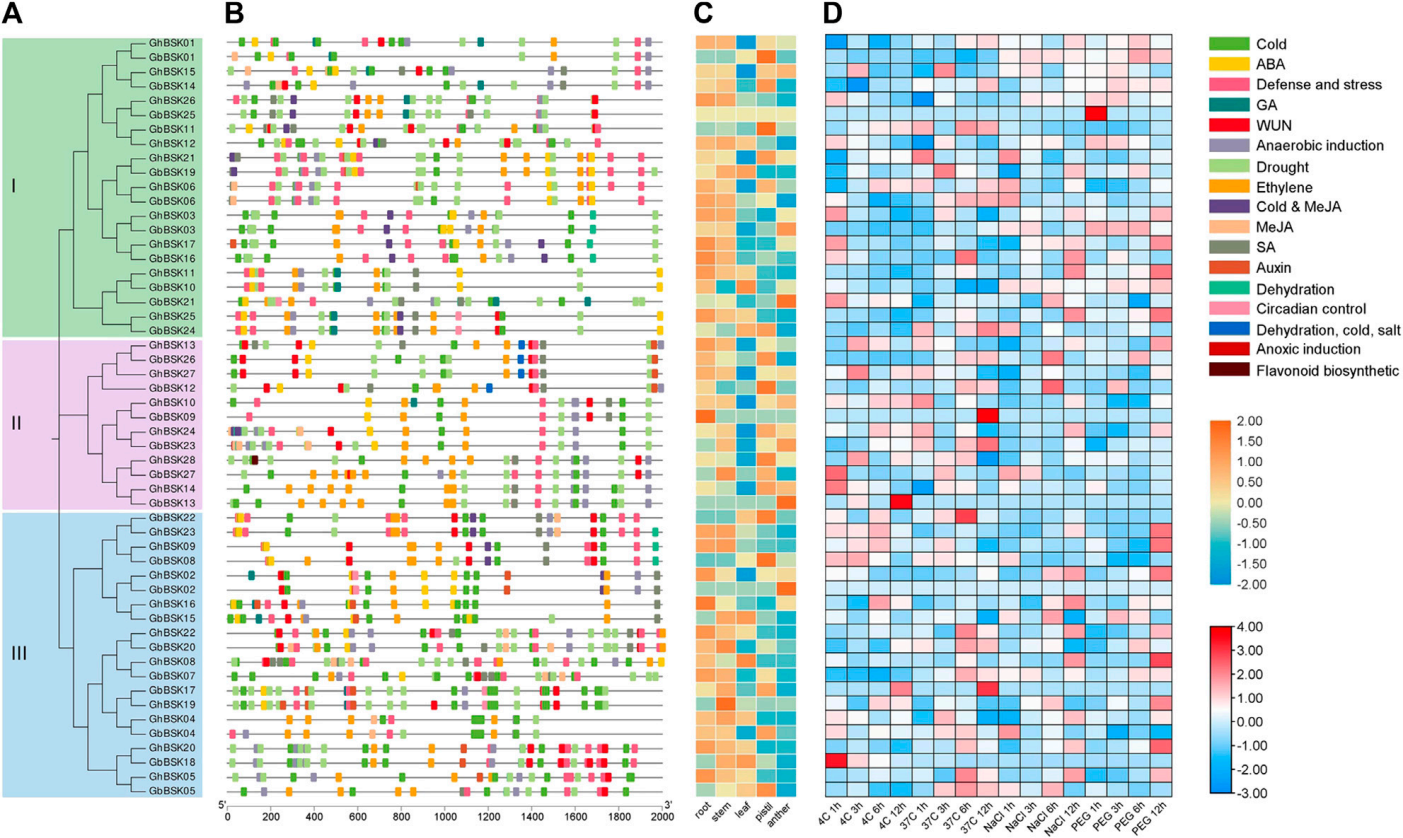
*Cis*-acting elements and expression pattern analysis. **(A)** Evolutionary relationship of *GhBSK* and *GbBSK*. **(B)** Prediction of *cis*-acting elements of *GhBSK* and *GbBSK*. **(C)** Expression pattern of *GhBSK* and *GbBSK* in different tissues. **(D)** Expression pattern of *GhBSK* and *GbBSK* under cold, heat, salt, and PEG. The values used for drawing the heat map were normalized FPKM.

*Cis*-acting elements analysis of cotton showed that BSKs had a large number of hormone-responsive elements and stress-responsive elements induced by cold and drought, indicating that BSKs may be regulated by various hormones at different growth stages and participate in the response regulation of cotton to various stresses (Figure 6). Tissue-specific expression analysis showed that BSKs were highly expressed in roots and stems, and some BSK I and BSK II genes were highly expressed in pistil. BSK III had a high express level in leaf. Gene expression analysis under cold, heat, salt and PEG stresses showed that BSKs were differentially expressed under different stress treatments, which were associated with stress response elements.

**FIGURE 7:**
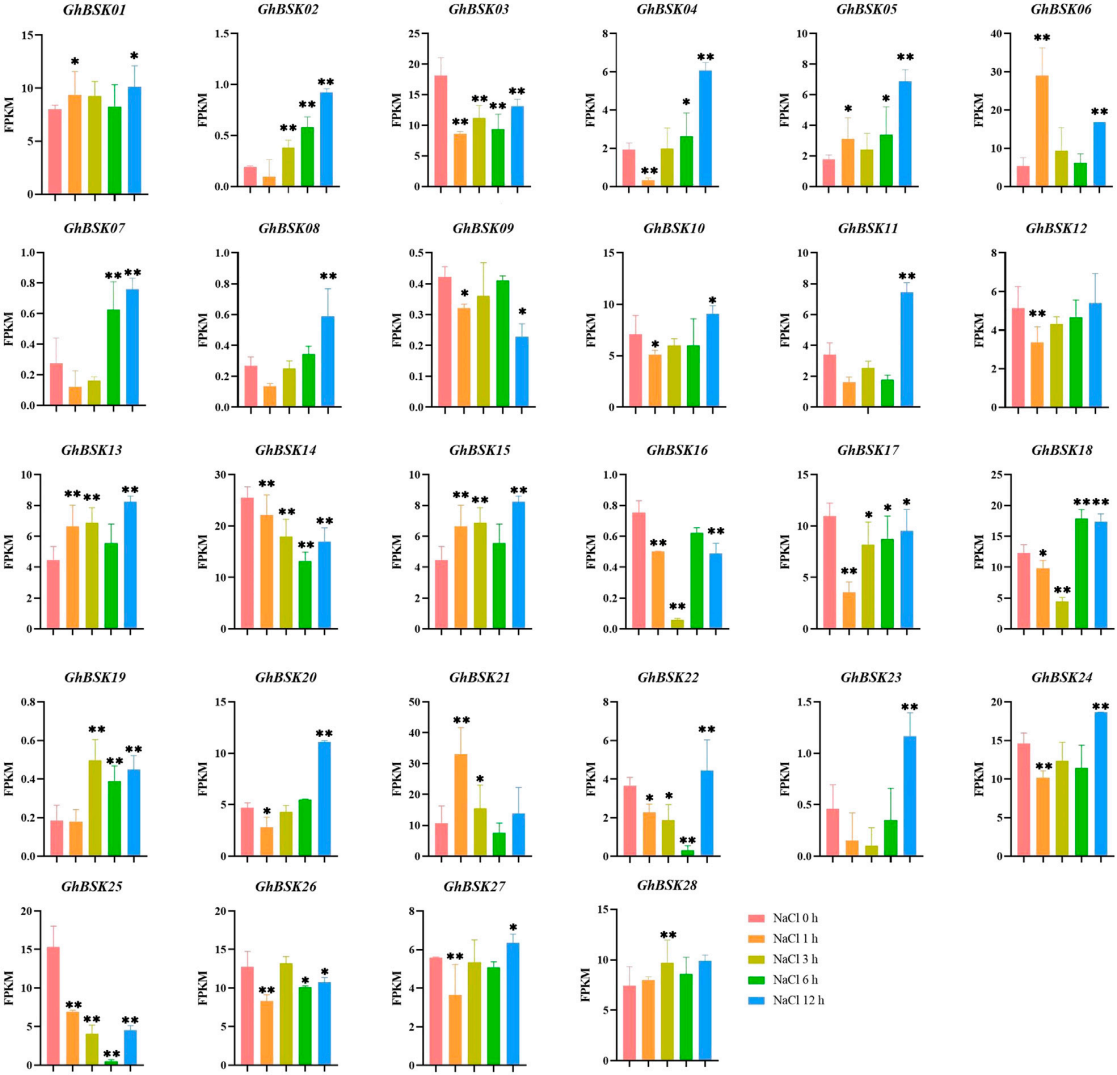
Expression analysis of *GhBSKs* under salt. The treatment groups were compared to NaCl 0 h group. Statistical significance was counted using one-way ANOVA. * mean *p* < 0.05, ** mean *p* < 0.01.

AtBSK5 and ZmBSK1 acted as a positive regulator in plant salt tolerance (Li et al., 2012; Liu et al., 2022). In order to explore the expression patterns of cotton BSKs under salt stress, the significance analysis of the expression levels of upland cotton BSK was carried out (Figure 7). The expression levels of *GhBSK03* and *GhBSK17* decreased significantly after salt stress and increased gradually with the increase of stress time. GhBSK06 and *GhBSK21* increased at 1 h after salt stress and then decreased, while *GhBSK06* increased again at 12 h after treatment. *GhBSK24* decreased at 1 h after salt stress and increased at 12 h after treatment. *GhBSK10* did not change significantly at the initial treatment but increased significantly 12 h after treatment. Taken together, it implies that 12 h is an intentional node, and some BSKs cannot respond to salt stress in time.

## 4 Discussion

Several studies have reported the roles of BSK proteins from *Arabidopsis* and rice in BR signaling and immunity, as well as in abiotic stress responses (Mora-García et al., 2004; Tang et al., 2008; Bayer et al., 2009; Li et al., 2012; Shi et al., 2013; Sreeramulu et al., 2013; Wang et al., 2017; Yan et al., 2018; Yu et al., 2018; Ren et al., 2019). However, the knowledge of BSK proteins in cotton is still quite limited. In this study, a total of 152 BSKs were identified in four cotton species and other 11 plant species by a genome-wide search, and bioinformatics methods were used to analysis their evolution, gene structure, promoter elements, protein structure and properties, gene expression patterns. Whole-genome duplication (WGD) has been reported in the two tetraploid cotton species studied (Paterson et al., 2012), and approximately two-fold amplification of the BSK gene was found. Genome duplication likely contributed the most to the expansion of the BSK gene family in many plant lineages. Approximately two-fold expansion of the BSK gene was found in plants with WGD, including *A. thaliana*, *P. trichocarpa*, and *Z. mays*. Furthermore, more than three-fold expansion of BSK was observed in *B. rapa*, possibly due to WTD (Li et al., 2019).

There are many similarities between cotton BSK genes. Except incomplete genes, BSKs possess an N-terminal PKc domain and a C-terminal TPR domain, consistent with previous studies (Tang et al., 2008). Although the expression patterns of BKSs were different, the homologous cotton BSKs proteins were basically identical in expression in tissues and organs, indicating that homologous proteins may have similar functions and redundancy.

The origin and development of the BR signaling system seems to be highly relevant with the evolution from aquatic to terrestrial plants, which have been observed in the ABA signaling system (Umezawa et al., 2010). A large number of ABA-responsive elements were also found in the promoter analysis of cotton BSKs (Figure 6). Besides ABA, a large number of hormone-responsive elements such as MeJA, GA, ethylene, SA and auxin were also found. It has been reported that BR interacts with ABA, Auxin, GA, JA and other hormones to regulate various growth and development processes of plants (Steber and McCourt, 2001; Assmann, 2004; Nemhauser et al., 2004; Perfus-Barbeoch et al., 2004; Hardtke et al., 2007; Finkelstein et al., 2008; Choudhary et al., 2012; Vandenbussche et al., 2013). BR is related to Auxin hypocotyl extension, root development, cell elongation, vascular bundle differentiation, lamellar tilt and shoot geotropism. Mutants of BR, which synergize with Auxin to promote cell elongation, often exhibit similar developmental defects, including an extremely short phenotype (Halliday, 2004; Teale et al., 2008). BR may affect plant stress response by promoting JA synthesis. BR also interacts with ethylene and auxin to regulate *Arabidopsis* shoot geotropism (Polko et al., 2013; Vandenbussche et al., 2013). Subcellular localization prediction of cotton BSKs revealed that no BSKs were anchored in the plasma membrane system.

Tissue-specific expression analysis of cotton BSKs suggests that different BSK proteins may function broadly in special tissues. The expression patterns of BSKs in the same subgroup were not always the same, and there were also differences in the expression patterns of *G. hirsutum* and *G. barbadense*. BR has been reported to play an important role in regulating root meristem maintenance, root elongation and promoting pollen germination and growth (González-García et al., 2011; Vogler et al., 2014). Significantly high expression of a large number of BSKs in the roots was observed in Figure 6. *GhBSK14*, *GbBSK01*, *GbBSK05*, *GbBSK08*, *GbBSK11*, *GbBSK12*, *GbBSK22*, and *GhBSK28* were only highly expressed in pistils, while *GbBSK02*, *GbBSK03*, *GbBSK13*, and *GbBSK21* were only highly expressed in anthers. Downstream genes of BSKs such as *Arabidopsis* BES1 and rice *OsBZR1* have been reported to be involved in anther and pollen development, revealing that BSK members may be involved in male gametophyte development (Ye et al., 2010; Zhu et al., 2015). BRs also regulate photomorphogenesis through BES1 and associate with light signaling (Wang et al., 2012; Wang et al., 2018). A large number of light-responsive *cis*-acting elements were found in cotton BSK family members, suggesting that they may have light-dependent transcriptional regulation. Lots of BSKs were repressed by cold, heat, salinity and drought, only a few escaped, which may be related to their low expression in leaves. Some genes showed broad responses to multiple stresses, such as *GbBSK05* was highly expressed under heat and salt stress; *GbBSK11* and *GbBSK23* were highly expressed under cold and salt stress, and *GbBSK01* was highly expressed under salt and drought stress, and a few genes only responded to one abiotic stress. The upregulated expression of individual genes at different time points indicated that cotton BSKs played a regulatory role in cotton growth, development, and stress response. Further research is required to identify the interacting proteins of cotton BSK and investigate the molecular mechanisms for improving plant salt tolerance. Furthermore, the study will explore the role of cotton BSK in resisting other abiotic stresses to cultivate multi-resistance cotton.

## 5 Conclusion

In this study, the BSK family members of *G. hirsutum*, *G. barbadense*, *G. arboretum* and *G. raimondii* were identified for the first time, and their evolutionary, gene structure, conservative structure and expression pattern were analyzed. The evolution of the BSK family gened were very conservative and were divided into three clades, with different distribution in different species. In cotton, the extended family is mainly replicated by the whole genome, without tandem repeat events. The expression of BSK gene is regulated by various environments, such as light, plant hormones and stress. In conclusion, GhBSKs and GbBSKs may play an important regulatory role in cotton growth and development and stress response, but how to regulate hormone and stress response through BR signaling pathway needs further experiments to prove.

## Data Availability

The original contributions presented in the study are included in the article/Supplementary Material, further inquiries can be directed to the corresponding author.
